# Patterns of Social Exclusion and Depression Levels Among Older Adults in South Korea

**DOI:** 10.1093/geroni/igaf122.1133

**Published:** 2025-12-31

**Authors:** Saemi Moon, Yeonjung Lee, Sulki Chung, Kyoungjae Park

**Affiliations:** Chung-Ang University, Seoul, Republic of Korea; Chung-Ang University, Seoul, Republic of Korea; Chung-Ang University, Seoul, Republic of Korea; Chung-Ang University, Seoul, Republic of Korea

## Abstract

This study examines the relationship between patterns of social exclusion and depression levels among older adults in South Korea, where rapid population aging coincides with high rates of poverty and suicide among people aged 65 and older. Older adults exhibit heterogeneous socioeconomic and health profiles, contributing to the distinct patterns of social exclusion. Social exclusion is significantly associated with individual well-being and societal cohesion. Thus, understanding complex interactions between the domains of exclusion is crucial. To examine these complex interactions, using data from the Korean Longitudinal Study of Aging in 2022, we analyzed a sample of 4,480 individuals who are aged 65 Latent Class Analysis identified four distinct patterns of social exclusion as followings: Income Exclusion; Social Relationship-Income Exclusion; Income-Health Exclusion; Multiple Exclusion. Results show that Income Exclusion was prevalent across all patterns, suggesting widespread and structural income-related challenges among older Korean adults. Additionally, people who aged 80 and older, with elementary education or less, and living alone were disproportionately represented in Multiple Exclusion group. Interestingly, urban residents showed higher levels of Social Relationship-Income Exclusion than rural residents, suggesting that urban environments may intensify social isolation despite greater service availability. Regarding the relationship between the social exclusion and depression, individuals who experience Multiple Exclusion show substantially higher depression than those with Income Exclusion. Findings demonstrate that exclusion across multiple domains significantly worsens mental health outcomes compared to single-domain exclusion, highlighting the importance of comprehensive approaches that address the interconnected nature of social exclusion dimensions rather than isolated interventions.

